# Comparison between Thermo-Alkaline and Electro-Fenton Disintegration Effect on Waste Activated Sludge Anaerobic Digestion

**DOI:** 10.1155/2019/2496905

**Published:** 2019-11-11

**Authors:** Emna Feki, Sami Sayadi, Slim Loukil, Abdelhafidh Dhouib, Sonia Khoufi

**Affiliations:** ^1^Laboratory of Environmental Bioprocesses, Centre of Biotechnology of Sfax, BP 1177, 3018, Sfax, Tunisia; ^2^Center for Sustainable Development, College of Arts and Sciences, Qatar University, Doha 2713, Qatar

## Abstract

Disintegration of municipal waste activated sludge (WAS) using thermo-alkaline (TA) and electro-Fenton (EF) methods was investigated and compared in terms of the efficiency of sludge solubilisation and enhancement of anaerobic biodegradability. Performance of organic matter solubilisation (soluble COD, proteins, polysaccharides) of sludge pretreated with EF was proved to be better than that with TA pretreatment, which resulted in the enhancement of anaerobic biodegradability. Comparison of results indicated that percentages of PN and PS release obtained after EF pretreatment (68.95 and 65.22%) were higher than those obtained by TA method (45.25 and 35.22%) respectively. An improvement of biogas potential about 2 and 1.6 times was achieved respectively by EF and TA pretreatment in comparison to raw sludge. During semi-continuous fermentation study in continuous stirred tank reactor, EF pretreated sludge gave the best biogas yield (0.6 L biogas/g COD) at an OLR of 2.5 g COD/L. d in comparison to TA pretreated sludge (0.3 L biogas/g COD), where low biogas yield about 0.1 L biogas/g COD was registered by raw sludge in the same CSTR. Therefore, the integration of EF process to anaerobic digestion might be a promising process for sludge reduction and biogas recovery.

## 1. Introduction

Domestic municipal wastewaters are widely treated by activated sludge process. This technology is an effective solution for returning clean and safe water back to its source; at the same time a huge amount of waste activated sludge (WAS) is produced. The generation rate of WAS is a function of the population number as well as the degree of treatment applied. Overall, an approximate average of 60 g/capita/day can be assumed [1]. For example, wastewater treatment plants (WWTPs) in Tunisia are expected to produce over 175000 dry metric tons in sludge each year [2]. The main method used for the management of WAS generated in Tunisian WWTPs is the natural evaporation using large evaporation ponds and then the in-situ storage. However, this method is found to have a negative impact on the environment due to the pathogen content and unstable organic matter nature.

The large quantities and increasing rates of worldwide production necessitate the development and application of good management approaches to sludge disposal. One of the challenges of sludge management is economic feasibility; sludge handling is responsible for about 30–40% of the capital cost of a treatment plant, and about 50% of the operating costs [3]. In response to this challenge, extensive researches have been conducted on the reuse of WAS for renewable energy production through anaerobic digestion [4]. On the other hand, the WAS anaerobic digestion rate is substantially limited by the first step of bioprocess, hydrolysis, which converts complex organic compounds into suitable substrates for methanogenesis. Commonly, it takes from 20 to 30 days to degrade 30% and 50% of raw WAS volatile solids, under optimum environmental conditions [5]. The slow hydrolysis rate of this kind of waste is due to the colloidal structure of sludge particulates, including the major constituting species of cells (proteins, carbohydrates, lipids, and volatile fatty acids) and extracellular polymeric substances (EPS) [6, 7]. Proteins are the major sludge compounds that represent about 50% of organic matter. The majority of these proteins (intracellular compounds) are protected from enzymatic hydrolysis by cell walls, but there is also a significant fraction of proteins in the Extracellular polymeric substances (EPS). These high molecular weight polymers play a significant role in floc stability, floc size, bioflocculation, and sludge settleability. But they are also regarded as one of the disadvantageous influences on sludge hydrolysis during anaerobic digestion [7].

In order to enhance biogas production and achieve faster degradation rate of WAS, various pretreatment techniques including chemical, mechanical, thermal, physical, and biological methods were largely studied [8]. In this context, over the last decade, researchers have proposed new ideas to intensify the WAS pretreatment by realizing intelligent combinations of established technologies, such as thermal-alkaline [9, 10], ultrasonic-Fenton [7, 11], electrochemical and sodium hypochlorite [12], and ozonation-microwave [13]. These processes have proven their effect on the acceleration of WAS hydrolysis and solubilisation of complex particulate matter by the disruption of sludge flocs, disintegration of bacteria cells and transfer of EPS and intracellular organic matters into the soluble fraction of the waste [8, 14].

Recently, the disintegration pretreatment by advanced oxidation processes have received extensive attention that can enhance biogas production, achieve faster degradation rate of WAS, and avoid potential environmental risk of WAS [8]. In this context, Fenton oxidation has been intensively applied for enhancing sludge dewatering, biogas production, and minimization of sludge weight [15, 16]. The efficiency of Fenton process is based on the generation of highly reactive radicals (•HO) that are unselective and powerful oxidizing species [17]. The generation in situ of hydroxyl radicals is due to the catalytic decomposition of hydrogen peroxide (H_2_O_2_) with iron ions (Fe^2+^) under the acidic condition [12]. These hydroxyl radicals can oxidize most organics into CO_2_, H_2_O, and inorganic ions via dehydrogenating or hydroxylating reaction [18]. Another well-established Fenton technology is the electro-Fenton (EF) approach, which relies on the electrochemical in-situ production of H_2_O_2_ or iron ions using specific electrodes. To date, most studies have focused on chemical Fenton pretreatment before anaerobic digestion, there is little information available concerning the use of EF pretreatment of WAS [11, 15, 19]. On the other hand, this technology seems to be one of the most promising advanced oxidation approaches to degrade pollutants retained in sludge, as residual pharmaceutical compounds, with no production of toxic intermediates by the strong oxidizing substances [20].

As such, the goal of the current study was to investigate the effects of applying an EF pretreatment on the performance of WAS anaerobic digestion. This was conducted by comparing the performance with a conventional disintegration method as thermo-alkaline (TA) pretreatment. The efficiency of sludge pretreatments on proteins and polysaccharides release, VSS solubilisation, and biogas yield was determined under the batch condition. The modified Gompertz model was used to evaluate the kinetic parameters and predict the biogas yield. Moreover, semi-continuous anaerobic fermentations of pretreated sludge using continuous stirred tank reactors (CSTR) were conducted. The performance of the reactors, biogas yield, and effluent characteristics was measured and compared to raw sludge.

## 2. Materials and Methods

### 2.1. Waste Activated Sludge and Inocula

Waste activated sludge (WAS) used for the disintegration and anaerobic digestion experiments was obtained from a municipal WWTP located in Sfax-Tunisia, which has a capacity of 215.000 eq.inh and a daily flow-rate around 17900 m^3^/d. The treated biological oxygen demand (BOD_5_) and chemical oxygen demand (COD) were 8800 Kg/d and 17597 Kg/d, respectively. Collected samples were stored at 4°C until use. The main characteristics of the raw and pretreated sludge are shown in Table 1.

The anaerobic microbial consortium (inocula) was obtained from an anaerobic digester installed in a WWTP located in Charguia city, Tunisia. The pH value of inocula was about 7.24. Total solid (TS), Volatile solid (VS), total suspended solid (TSS), and volatile suspended solid (VSS) concentrations were about 20.1, 11.23, 15.31, and 9 g/L, respectively.

### 2.2. WAS Pretreatment

A glass reactor with working volume of 300 mL was used for thermo-alkaline pretreatment. NaOH solution (5N) was used to adjust pH of sample to a value around 10. After that, sample was homogenized for 24 h at ambient temperature (28°C) and 2 h at 105°C. These conditions were optimized in a previous study [21].

Electro-Fenton pretreatment was carried out in a glass reactor connected to an electric generator ASF type 400/40.10. Two iron electrodes which were positioned approximately 2.5 cm apart from each other were used. The active surface area of electrodes was 0.16 dm^2^. Electrolysis experiments were operated in batch mode by treating 300 ml of raw WAS. The pH of WAS sample was adjusted to 3 by adding HCl solution (2N). Current density, reaction time, and H_2_O_2_ concentration were fixed, respectively, at 2.5 A/dm^2^, 1 h, and 1.8 g/l as optimum conditions determined in previous study. During electrolysis treatment, continuous homogenization was maintained to avoid sedimentation of sample.

The disintegration degree after sludge pretreatment was evaluated by determining VSS solubilisation. The solubilisation percentage was calculated according to this formula [5]:

(1) $$\begin{array}{c} VSS\;solubilisation\;yield\left(\%\right)=\left(\left(VSSt0-VSStf\right)\times \frac{100}{VSSt0}\right) \end{array}$$

With VSSt0 as the VSS before treatment and VSStf as the VSS after treatment.

### 2.3. Batch Anaerobic Digestion

Batch fermentation assays under mesophilic conditions (37 ± 1°C) were conducted to evaluate the biogas production from raw and pretreated sludge samples. Sealed 120 mL serum bottles were used as anaerobic reactors. A working volume of 60 mL was kept in all batches. WAS sample (substrate) and inocula were introduced to the reactor with the same proportion keeping a VS substrate/VS inocula ratio equal to 1 [22]. The pH of anaerobic medium was adjusted to 7.2. To supply anaerobic condition, all batches were purged with a gas mixture of 75% N_2_ and 25% CO_2_ for 2–3 min. Control batch was conducted only with inocula in order to estimate the biogas production resulting from the seeding material. All anaerobic batch tests were conducted in duplicate.

Daily biogas production was measured using a gas displacement device. Biogas yield was calculated in terms of biogas volume per mass of substrate VS introduced at the initial time of fermentation. The fermentation tests were conducted for approximately 30 days until biogas production stopped. The modified Gompertz equation was used to study the cumulative biogas generation from batch digesters and the kinetics of biogas production [23]. It is a function of bacterial growth. The modified Gompertz equation is given by:

(2) $$\begin{array}{c} \left(M=P.\text{exp}\left\{-\text{exp}\left[\frac{\text{Rm}\times e}{P}\left(\lambda -t\right)+1\right]\right)\right\} \end{array}$$

where *M* is the cumulative biogas production (L) at any time (*t*), *P* the biogas yield potential (L/g VS), Rm the maximum biogas production rate (L/g VS. d), *λ* the duration of lag phase (day), and *t* is the duration of the assay at which cumulative biogas production *M* is calculated (day), and *e* the exp(1) = 2, 7183. The parameters *P*, *Rm* and *λ* were estimated for each of the digesters using MATHEMATICA model.

### 2.4. Semi-Continuous Anaerobic Digestion

A continuous stirred tank reactor (CSTR) with a working volume of 1.5 L was used to conduct two semi-continuous fermentations. The reactor was initially fed with raw sludge for 20 days then with TA pretreated sludge until the end of the first fermentation (F1). During the second fermentation (F2), the reactor was fed with EF pretreated sludge for 80 days. The digester was operated at 37°C and stirred continuously at 200 rpm. Sludge retention time (SRT) ranging between 30 and 15 days was maintained which is in correlation with the applied organic loading rate (OLR). Every day, digested sludge was drained and the same volume of feed sample was fed to the reactor. Biogas production was measured by displacement of liquid. It should be noted that the pH of feed sample was corrected when its value is not around 7.

### 2.5. Physico-Chemical Analytical Methods

Characterization of sludge samples was performed by determining the following parameters: pH, soluble chemical oxygen demand (soluble COD), total chemical oxygen demand (total COD), Biological oxygen demand (BOD_5_), total solids (TS), volatile solids (VS), total suspended solids (TSS), volatile suspended solids (VSS), total kjeldahl nitrogen (TKN), proteins (PN), and polysaccharides (PS). Details of methods used for the determination of these parameters were mentioned in previous study [21].

Concentration of PN was determined by using the Bradford method [24]. 800 *µ*l of the diluted sample containing 0–10 *μ*g of protein per ml is mixed with 200 *μ*l of Biorad reagent. The mixture was maintained at ambient temperature and in the dark during 10 min. The optical density was determined at 595 nm. The protein concentration in each sample was determined by using a calibration curve prepared with concentrated bovine serum albumin (BSA) solution.

PS concentration was analyzed using the Dubois method [25]. 200 *μ*l of sample, 200 *μ*l of phenolic solution (5%), and 1 ml of concentrated H_2_SO_4 _were mixed and taken to a water bath at 100°C for 5 min. After that, samples were allowed to cool for 30 min in the dark and then photo-metrically analyzed at 485 nm. Results were determined by using a calibration curve prepared with glucose solution.

## 3. Results and Discussion

### 3.1. TA and EF Pretreatment of WAS

The expected effect of TA and EF pretreatment on sludge was the release of organic materials, with interest focused on solubilisation of COD, polysaccharides, proteins, and VSS, thus enhancing hydrolysis in order to improve biogas potential of sludge. Table 1 showing the characteristics of sludge samples indicates the improvement of EF pretreated sludge quality for subsequent biological treatment in comparison to raw and TA pretreated Sludge. Soluble COD (4 g/L) and NTK (2.61 g/L) concentrations were higher in EF pretreated sample in comparison to raw and TA pretreated WAS. It can be noted that EF could contribute to the solubilisation of sludge, but it did not degrade the organic compounds greatly [26]. The pH of EF pretreated sludge was increased to value in the range of neutrality, which is favorable for anaerobic post-treatment. Indeed, in the case of TA pretreatment, an alkaline pH was obtained which requires a correction prior biological treatment. The increase of soluble COD for the both pretreatment methods indicated that they have the potential to damage excess sludge structure and cell membranes and to release extracellular and possibly intracellular compounds with high solubility. EPS are composed of proteins (PN) and polysaccharides (PS), and the solubilisation of these compounds reflects the disintegration degree of sludge [27]. In this study, proteins, polysaccharides, and VSS concentrations were considered the main parameters for evaluation of sludge disintegration.

#### 3.1.1. Effect on Proteins and Polysaccharides Release

**Figure 1:**
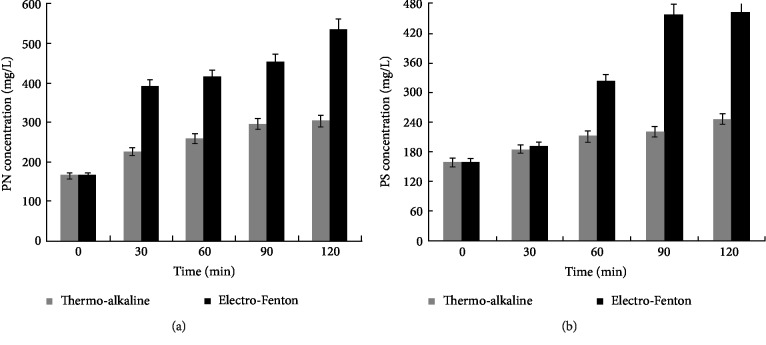
Evolution of proteins (PN) (a) and polysaccharides (PS) (b) concentration during EF and TA pretreatment.

The evolution of PN and PS concentration during TA and EF pretreatments are shown in Figure 1. Results show that the concentration of these compounds in the soluble fraction increased with the increase of EF and TA treatment time. In fact, the PN concentration in raw sludge was low (166.62 mg/L). It increased to 304.31 and 536.62 mg/L after TA and EF pretreatment, respectively. The same result was obtained for PS where the concentration increased from 160 to 247 and 460 mg/L, respectively, in TA and EF pretreated sludge. The increase of PN and PS concentration in the pretreated sludge could be due to the cell lysis and/or solubilisation of EPS. So, that there is more solubilisation of organic matters observed by the increase of soluble COD (Table 1). Comparison of results indicated that percentages of PN and PS release obtained after EF pretreatment (68.95 and 65.22%) were higher than those obtained by TA method (45.25 and 35.22%) respectively. Furthermore, a rapid release of PN and PS in the case of EF treatment was observed which indicated the strong effect of electro-chemical reactions on the disruption of sludge particulates. This could be explained by the effect of free radicals generated during EF reaction which led to the oxidative degradation of EPS. Indeed, Yuan et al. [28] showed that after sludge disintegration with electrolysis (anode Ti/RuO_2_), the EPS and intracellular substances are released into the aqueous phase and which cause the increase of the concentration of PN and PS. Also, it has been reported that electrochemical pretreatment can convert high molecular weight biopolymer substances to low molecular weight products [17, 29]. In fact, solubilisation of PN and PS by the two methods can make them more accessible to microorganisms during a biological post-treatment.

#### 3.1.2. Effect on VSS Solubilisation

**Figure 2:**
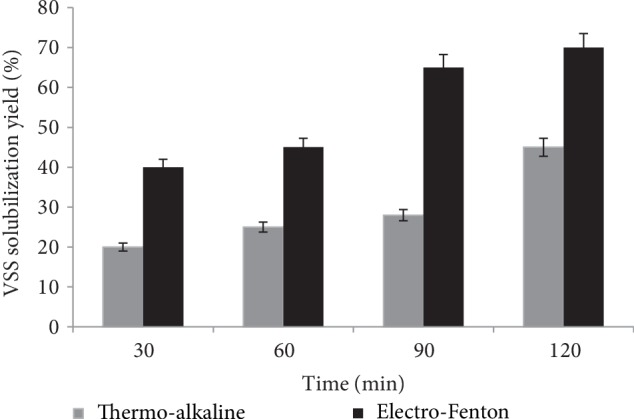
VSS solubilisation yield during EF and TA pretreatment.

The solubilisation rate of the particulate volatile suspended solids (VSS) was often used to evaluate the impact of pretreatment on the sludge hydrolysis. Results are summarized in Figure 2. An increase of VSS solubilisation percentage (%) was observed at treatment time up to 120 min. At this time, VSS solubilisation percentage after TA and EF pretreatement was 45% and 70%, respectively. It was clear that EF pretreatment has given the highest VSS solubilisation compared to TA treatment. This was accredited to the high activity of hydroxyl radicals generated during EF reaction which caused the cleavage of the cell walls of the sludge biomass and the release of intracellular materials into the aqueous medium phase.

To characterize the organic fraction distribution of sludge, VSS/VS ratio was determined after sludge pretreatment. Results are shown in Table 1. The VSS/VS ratio of raw sludge was approximately 0.87, in which the concentration of TSS is high (14.67 g/l). After TA and EF pretreatment, this ratio has decreased to 0.47 and 0.31, respectively. Thus, the pretreatment induced an important solubilisation of organic matter proved by the decrease of VSS/VS ratio [5]. These results mean that the sludge was effectively solubilized and organic molecules were released from suspended organic fraction. The TSS reduction after pretreatment was due to the lysis of cells, but the reduction is most important in the case of EF pretreatment. The difference of VSS/VS ratio between both pretreated sludge noted that during EF pretreatment there was a degradation and mineralization of organic molecules proved by the decrease of the VSS (9.15 g/l) [30]. From these results, it can be concluded that EF process can achieve a best degree of disintegration in comparison to TA pretreatment. The efficiency of this process to improve sludge disintegration was also reported by Alzadeh Fard et al. [31].

### 3.2. Anaerobic Digestion in Batch Condition: Biogas Potential

**Figure 3:**
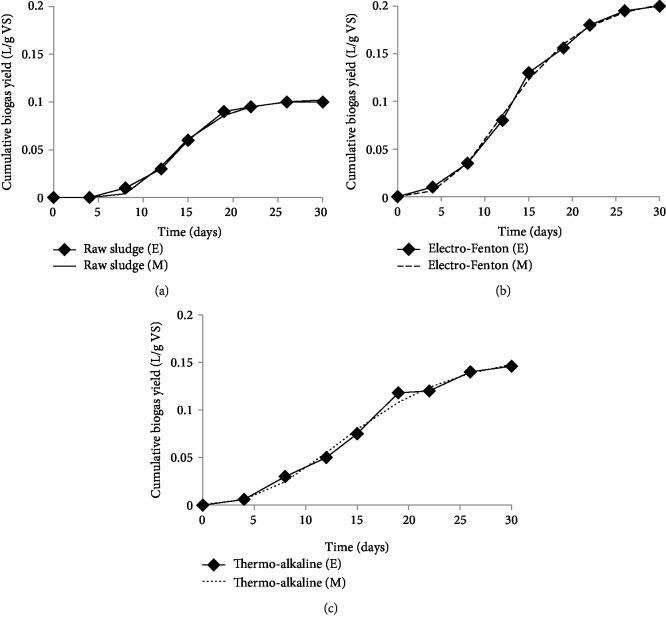
Cumulative biogas yield of raw sludge (a), EF (b), and TA (c) pretreated sludge. (E) Experimental data; (M) Modified data using modified Gompertz equation.

TA and EF pretreatments have been proven in the first part of this study for their effect on the solubilisation of sludge. However, this parameter cannot be directly related to an improvement of anaerobic digestion in terms of biogas production [32, 33]. To reveal the feasibility of using the studied pretreatments to enhance anaerobic digestion, batch fermentation assays were performed with raw and pretreated sludge under mesophilic conditions. Cumulative biogas yield in serum bottles was monitored for 30 days of fermentation (Figure 3). The ultimate biogas yields were calculated by fitting experimental data with the Gompertz equation. The kinetic constants were estimated using nonlinear regression and are summarized in Table 2.

During the first period of fermentations, biogas production has increased slowly due to the lag phase of microbial growth where microorganisms need an acclimation period under the new environmental conditions. After the lag phase, the biogas production starts to increase progressively with time fermentation due to exponential growth of the microorganisms, which leads to faster degradation of substrate.

The evolution of biogas production for control sludge showed a lag phase (*λ*) about 8.69 days and production increased until day 18 to reach a value of 0.1 L/g VS and no significant increase was observed later. The extended lag phase of the control could be explained by the slow hydrolysis of raw substrate. However, a shorter lag phase (*λ*) was exhibited by TA and EF pretreated sludge at 5.37 and 5.56 days, respectively. The early exponential biogas production during fermentation of pretreated samples makes evidence the ease of metabolizing the substrates and the availability of biodegradable substances. These aspects were more facilitated by EF than TA. Thus, EF pretreated sample gave the highest biogas yield (0.2 L/g VS) and TA pretreated sample had a biogas yield of (0.16 L/g VS). This result notes the improvement of sludge biodegradability after EF pretreatment in comparison to TA pretreatment and an increase of biogas yield about 2 times was registered compared to raw sludge. This result is in accordance with the study of Yu et al. [26] that demonstrated the affectivity of electrochemical pretreatment for the enhancement of biogas yield in comparison to thermal-alkaline methods. In other recent study conducted in pilot scale, the biogas productivity of sludge pretreated with electrochemical and sodium hypochlorite combination method was increased by 1.83 times compared to that untreated sludge [12]. Also, to enhance the anaerobic digestion of secondary sludge Fenton pretreatment (60 g H_2_O_2_/Kg TS, 0.07 g Fe^2+^/g H_2_O_2_, and pH 3) was studied [15]. In this study, researchers noticed 15% increase in methane yield, 3.1 times increase in net energy as well as considerably reduced GHG emission. Compared with the results of these previously reported studies, the EF pretreatment in the present study was more effective in enhancing biogas production of sludge anaerobic digestion. The improvement of biogas production with EF pretreatment showed a strong indication that pretreatment converted a portion of the non-biodegradable materials to be easily available to the fermenting bacteria.

### 3.3. Anaerobic Digestion in Semi-Continuous Reactor

**Figure 4:**
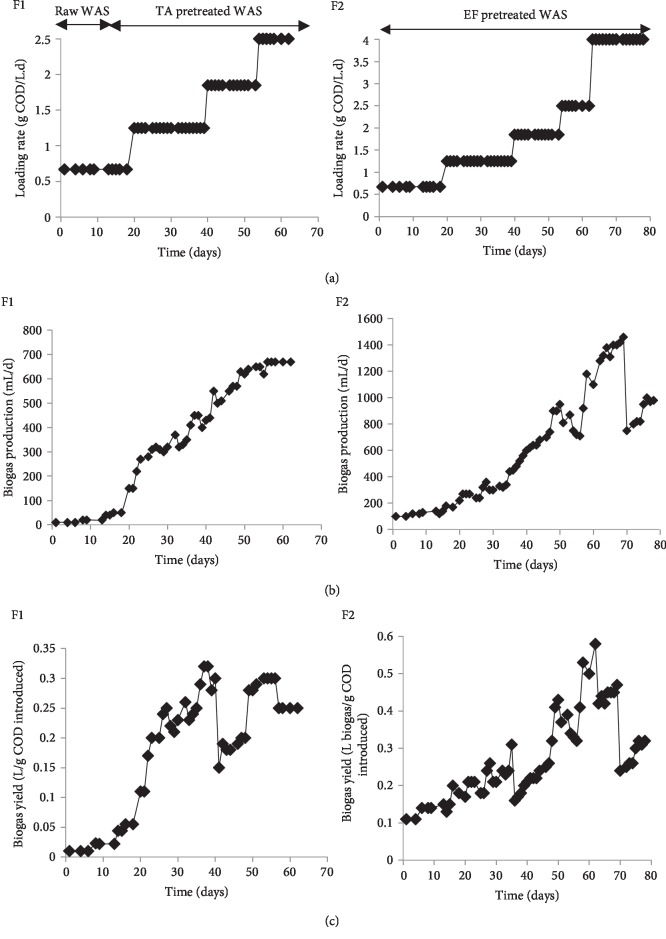
Evolution of organic loading rate (a), biogas production (b), and biogas yield (c) during semi-continuous anaerobic fermentation F1 (raw and TA pretreated sludge) and F2 (EF pretreated sludge).

Figure 4 shows the evolution of the organic loading rate (OLR) and biogas production and yields during semi-continuous fermentations (F1 and F2) of raw and pretreated sludge in CSTR. At the beginning of fermentation F1 (1–15 days), the reactor was fed with raw sludge at an OLR of 0.6 g COD/ L. d (Figure 4F1(a)) then the feeding was changed by TA pretreated sludge until the end of experiment. Low biogas production was registered during raw sludge digestion and yields did not exceed 0.05 L biogas/g COD (0.02 L biogas/g VS) which are lower than those (0.25 L biogas/g VS) found by Nges and Liu. [34]. This can be explained by the difficulty of floc disintegration and cell bacterial lysis of secondary sludge [5]. In fact, a disintegration step before anaerobic fermentation can improve the conversion of organic matter into biogas. This was demonstrated by feeding the reactor with TA pretreated sludge. Indeed, a gradual increase of OLR was applied to digester that reached 2.5 g COD/L. d at the end of F1. As a result, a significant increase of biogas production was observed and a volume up to 420 mL/d was registered at an OLR of 1.2 g COD/L. d (Figure 4F1(b)). This finding proved the improvement of biogas production with pretreated sludge in comparison to raw sludge. A correlation between the biogas production and the OLR applied to reactor was also observed. The best biogas yields (0.3 L/g COD introduced) were obtained at OLRs between 1.2 and 1.8 g COD/L. d (Figure 4F1(c)).

Carrere et al. [35] investigated the TA pretreatment before the co-digestion of waste-activated sludge and fatty wastewater in semi-continuous reactors and stated that this pretreatment led to a significant increase of methane production (+58%). The same results were obtained by Kim et al. [32, 33]. Amelioration of biogas production about 73.9% was obtained during AD of sludge hydrolysed with 0.10 M NaOH at 73.7°C during 6 h. Furthermore, Xu et al. [36] showed an increase of 34% in the biogas production by treating sludge by TA method (pH 11 at 90°C during 10 hours). For industrial application, thermo-alkaline method was proven to be a technically and economically feasible method for sludge hydrolysis before anaerobic digestion [37].

**Figure 5:**
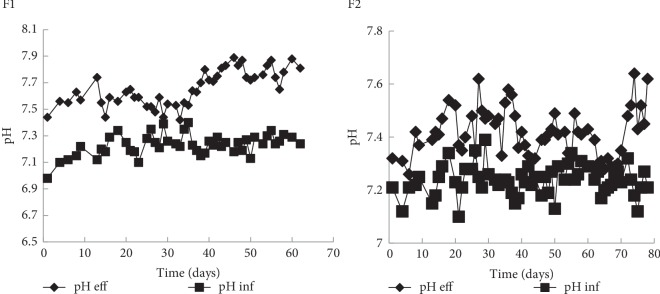
Evolution of influent and effluent pH during the semi-continuous anaerobic fermentation F1 and F2.

All of the above results show that the biogas yield of TA pretreated sludge was improved during the semi-continuous fermentation and this indicates the stability of anaerobic system using CSTR (Figure 5). The same results were obtained when the digester was fed with EF pretreated sludge (F2). As shown in Figure 4F2(a), an OLR 0.6 g COD/L. d was applied at the beginning of F2. At 20^th^ day, OLR was increased to 1.25 g COD/L. d and then gradually increased with the fermentation time to reach 4 g COD/L. d (63^th^ day).

Figure 4F2(b) shows the evolution of biogas production during this fermentation. The biogas production started to increase from the first day of F2 which could be explained by the availability of substrate and the easy biodegradation. A maximum biogas volume of 1460 mL/day was obtained at an OLR of 2.5 g COD/L. d. which resulted to a biogas yield about 0.6 L biogas/g COD. However, a slight decrease of biogas yield was observed with the increase of OLR to 4 g COD/L. d. These results have confirmed that the EF pretreatment enhances the anaerobic digestion. An increase of biogas yields about 2 and 12-fold was registered in comparison to TA pretreated (0.3 L/g COD) and raw sludge (0.05 L/g COD), respectively. The biogas yield of EF pretreated sludge was considered important. This result could be explained by the fact that EF pretreated sludge contained higher concentration of soluble organic matter which microorganisms were able to utilize immediately. Another factor that can be added is the impact of iron (solubilized during EF pretreatment) on anaerobic fermentation, which is considered as an essential element and a potential electron donor [38]. The stimulating effects of iron on microorganisms and enzyme activities were elaborated by Wei et al. [39].

**Figure 6:**
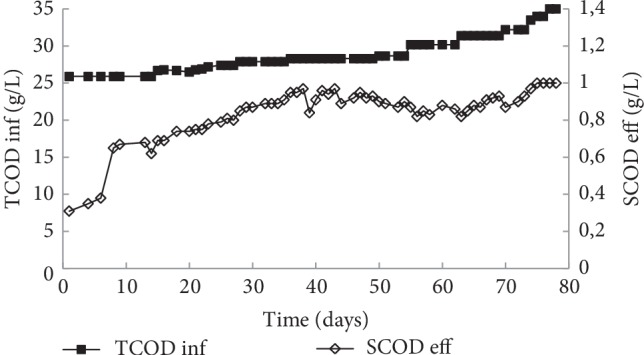
Evolution of influent and effluent COD during the semi-continuous anaerobic digestion of EF pretreated WAS.

Figure 6 gives the evolution of total COD of EF pretreated sample used for reactor feeding and soluble COD of digestate. It is noted that total COD concentration was between 25 and 35 g/L. While for the soluble COD of digestate, values seem very low at the beginning of fermentation then gradually increased with the increasing of OLR. A stability of COD removal of 82.53% (data not shown) was noted during the application of an OLR of 4 g COD/L. d. In fact, the performance of COD removal in the reactor was in correlation with the initial COD influent concentration, the SRT, and the biological consortium activity. The high COD removal could be explained by the increase of biodegradability activity of the anaerobic consortium in the presence of EF pretreated sludge [40].

## 4. Conclusion

In this study, EF and TA pretreatments were explored for improving AD performance of WAS. The results indicated that the two methods have the potential to damage sludge structure and cell membranes and to release extracellular and possibly intracellular compounds with high solubility. However, the highest organic matter solubilisation proven by the release of PN, PS, and soluble COD was registered by the EF method. This resulted to the increase of WAS components bioavailability under batch and semi-continuous mesophilic fermentation. An increase of biogas yields about 2 and 12-fold was registered in comparison to TA pretreated and raw sludge, respectively. So, the solubilisation and anaerobic digestibility results for the EF process is satisfactory to improve biogas potential of WAS. In order to evaluate the techno-economic feasibility of the combined technology EF-AD, a pilot scale process will be developed in a future study.

## Figures and Tables

**Table 1:** Characteristics of waste activated sludge before and after thermo-alkaline (TA) and electro-Fenton (EF) pretreatments.

Parameters (g/L)	Raw	TA pretreated	EF pretreated
pH	6.95 ± 0.2	9.7 ± 0.5	7.57 ± 0.12
TS	18.64 ± 1.6	16.17 ± 5.9	12.48 ± 6.31
VS	10.84 ± 1.3	11.23 ± 1.38	9.15 ± 4.75
TSS	14.67 ± 1.6	14.15 ± 0.17	11.32 ± 0.08
VSS	9.5 ± 1.3	5.23 ± 0.05	2.85 ± 0.74
Total COD	18.10 ± 2	16.86 ± 0.27	14.5 ± 4.71
Soluble COD	1.2 ± 0.5	3.3 ± 0.65	4.1 ± 1.4
NTK	1.91 ± 1	1.98 ± 0.38	2.61 ± 0.86
COD/NTK	9.45	5.83	3.83
VSS/VS	0.87	0.47	0.31

**Table 2:** Kinetic parameters calculated from the theoretical model of raw and pretreated sludge.

Samples and treatment	Modified Gompertz parameters (model)			*R* ^2^	Rmsd
	*P* (L/g VS)	Rm (L/g VS.d)	*λ* (day)			
Raw sludge	0.1032	0.0099	8.6975	0.921	0.0036
Thermo-alkaline	0.1611	0.0083	5.3752	0.966	0.0015
Electro-Fenton	0.2098	0.0133	5.5618	0.986	0.0006

## Data Availability

The data used to support the findings of this study are included within the article.

## Abbreviations

AD: Anaerobic digestion
CSTR: Continuous stirred tank reactor
COD: Chemical oxygen demand
Soluble COD: Soluble chemical oxygen demand
Total COD: Total chemical oxygen demand
EPS: Extracellular polymeric substances
PN: Proteins
PS: Polysaccharides
TKN: Total kjeldahl nitrogen
TS: Total solids
TSS: Total suspended solids
VS: Volatile solids
VSS: Volatile suspended solids
WWTP: Wastewater treatment plant.

## Additional Points

*Highlights.* (i) Thermo-alkaline and electro-Fenton methods were investigated for the disintegration of waste activated sludge. (ii) Electro-Fenton method was highly efficient in solubilisation of sludge. (iii) The anaerobic digestion of pretreated sludge was investigated under batch and semi-continuous conditions. (iv) An improvement of biogas potential was achieved by electro-Fenton and thermo-alkaline pretreatment. (v) Biogas production during semi-continuous fermentation was comparatively high in electro-Fenton method.
